# Sensorial evolution of cassava flour (*Manihot esculenta* crantz) added to protein concentrate cassava leaves

**DOI:** 10.1002/fsn3.16

**Published:** 2012-12-26

**Authors:** Elaine C S Lima, Márcia B S Feijo, Maria C J Freitas, Edna R dos Santos, Armando U O Sabaa-Srur, Luciana S M Moura

**Affiliations:** 1 Post Graduation Program Science and Food Technology of Federal Rural University of Rio de Janeiro, Rio de JaneiroRJ, Brazil; 2 Institute of Nutrition of Josue de Castro, Federal University of Rio de JaneiroRio de Janeiro, RJ, Brazil; 3 The Brazilian Society of Fruits and Vegetables, Rio de JaneiroRJ, Brazil

**Keywords:** Acceptance test, protein concentrate, sensorial

## Abstract

Cassava is regarded as the nutritional base of populations in developing countries, and flour, product made of cassava, is the most consumed in the world. The cassava leaves are very rich in vegetable proteins, but a big amount is lost in processing the crop. The objective of this study was to do a sensory evaluation of cassava flour to which a protein concentrate obtained from cassava leaves (CPML) was added. The CPML was obtained from cassava leaves by isoelectric precipitation and added to cassava paste for preparation of flour in three parts 2.5, 5, and 10%. The acceptance test was done by 93 consumers of flour, using hedonic scale of 7 points to evaluate characteristics like color, scent, flavor, bitterness, texture, and overall score. By the method of quantitative descriptive analysis (QDA), eight trained tasters evaluated the following characteristics: whitish color, greenish color, cassava flavor, bitter flavor, characteristic flavor, lumpiness, raw texture, leaf scent, and cassava scent. The acceptability test indicated that flour cassava with 2.5 was preferred. Whitish color, greenish color, cassava flavor, bitter flavor, salty flavor, characteristic flavor, lumpiness texture, raw texture, and the smell of the leaves and cassava flour were the main descriptors defined for flour cassava with CPML has better characteristics.

## Introduction

The cassava is considered an alimentary base for people in Africa, Asia, and Latin America assuming socioeconomical position in the world, due to its high capacity to adapt to climatic conditions. Its roots can be easily cultivated and it is the principal source of carbohydrates for needy people with protein deficiency, which is one of primary factors of human malnutrition that affects a big part of population. This deficiency can be the result of lack of protein containing foods, of both animal and vegetative origin, and its high price.

This vegetable is characterized by its high concentration of carbohydrates that is why it is considered a caloric aliment. The cassava roots also contain vitamin C, carotenoids, thiamine, riboflavin, and nicotinic acid. They also represent considerable quantities of calcium and phosphorus (NEPA/UNICAMP 2006).

The cassava leaves have been examined in Brazil and different countries due to its nutritional characteristics and big waste in the field (Modesti 2006). High levels of protein in the cassava leaves were mentioned in different works, with its variety from 20.77 to 35.9 g/100 g (Madruga and Câmara 2000; Ortega-Flores et al. 2003; Wobeto 2003).

The utilization of biomass became a world concern, especially in Brazil, the country, which is rich in variety and quantity of foods. However, thousands of Brazilians are still starving, while a big part of biomass is wasted as in the case of subsistence plants, and the cassava (*Manihot esculents* Crantz) is one of them. During the harvest, it has its aerial part left in the field (Ferri 2006). The leaves contain important nutrients which could be used in a diet as a protein portion (Corrêa et al. 2004).

Sensorial evaluation is a very important tool to evaluate new dietary products because it helps to obtain aliments that are pleased a consumer. Hence, the objective of this work was to evaluate the addition of protein concentrate cassava leaves (CPML) to cassava flour by acceptance test to consumer level and by quantitative descriptive analysis (QDA) with trained tasters.

## Material and Methods

### Obtaining of cassava flour with addition of CPML

The CPML was obtained from cassava leaves by isolectric precipitation (Lima et al. 2011). Processing of cassava leaves with CPML consisted of the following steps: selection of cassava leaves, washing to remove the dirt, manual peeling, washing in the running water, sanitation, triturating, pressing, adding of CPML to mass in three concentrations (2.5, 5.0, and 10%), drying in ventilated stove at 60°C, milling, screening, roasting with homogenization with manual mixer, cooling, and filling. Therefore, four formulations of cassava flour were produced: 0.0% CPML – F_0_; 2.5% CPML – F_1_; 5.0% CPML – F_2_, and 10% CPML – F_3_.

This sensorial analysis study was previously approved by The Committee of Ethics of Federal University of Rio de Janeiro (UFRJ), No. 069/2010.

### Acceptability test

The acceptance test was done with one sample of cassava flour without CPML (control) and three samples of cassava flour with 2.5, 5, and 10% CPML, respectively. There were 93 nontrained tasters in completely randomized groups, 10 g of encoded samples with three digits, accompanied with one glass of water at room temperature to clean the mouth and tongue before each evaluation (Dutcosky 2007).

To study the acceptance of the cassava flour, a hedonic scale of 7 points was used (7 = liked a lot and 1 = disliked a lot) to evaluate appearance, scent, flavor, texture, and overall score. The hedonic results were expressed in graphics of frequency distribution and variety analysis (analysis of variance [ANOVA]) and comparative test of averages (Turkey), according to Stone (1994) and Stone and Sidel (1992) using statistic program XLSTAT, estimation error with of 5%.

### Quantitative descriptive analysis

A QDA test was used to characterize and quantity the sensory attributes of control flour and with 2.5, 5, and 10% CPML, respectively, was determined using the technique of QDA, according to Stone (1994). The tests were performed at the laboratory of Aliments Analysis and Processing, in the Nutrition Institute of Josue de Castro of UFRJ, which constructions include individual cabins and illumination control.

#### Recruiting of tasters

There were recruited 16 individuals among students and employees of the Nutrition Institute of Josue Castro of UFRJ. The criteria to form a team were to be a consumer of cassava flour and to be selected in teams by selective tests as its sensorial perceptiveness is normal (recognition of basic flavors and smells).

#### Development of descriptive terminology

The following products were chosen to establish descriptive terms: distillated water, wheat flour, toast, lemon gelatin powder, cooked cassava, 0.06% solution of caffeine in water, cookies cream cracker, corn flour, and cassava leaf.

The products were served in disposable materials, encoded with numbers of three digits, containing 10–20 g of each product. Panelists were asked to describe the characteristics of the samples related to the color, flavor, texture, and smell. Next, a list of all used terms and number of times that they were reported was prepared. This led to discussions in groups with the objective of creating descriptive terms and reference samples of scale extremes.

The result of four evaluation sessions of reference samples and discussions in group was the evaluation list of control flour and flour with 2.5, 5, and 10% CPML.

#### Panelist selection and descriptive analysis of samples

The samples were analyzed initially by 16 panelists, in triplicate, in four sessions in subsequent days. Cards for recording descriptions were used by all team members. Each descriptive term was evaluated in an unstructured line scale, anchored at extremes with the following terms of intensity: strong and weak. After the panelists had completed their test, the distance from the left end of the line to the point marked by the panelist was measured.

The flour was served in paper cups, encoded with numbers of three digits, accompanied by a glass of water at room temperature. The tests were done as open trials. ANOVA was done on the results of each taster for every evaluated characteristic, considering variation point samples and repetition. The final descriptive panel was chosen, those panelist that represented discriminative ability *P*_sample_ < 0.05, good reproducibility at trials (*P*_repetition_ > 0.05), and accordance with other members. So, the team was formed of eight tasters.

The obtained data were submitted to variance analysis (ANOVA) and comparative test of averages (Turkey), estimation error with of 5%, according to Stone (1994) and Stone and Sidel (1992) using statistic program XLSTAT.

## Results and Discussion

### Acceptability test

**Figure 1:**
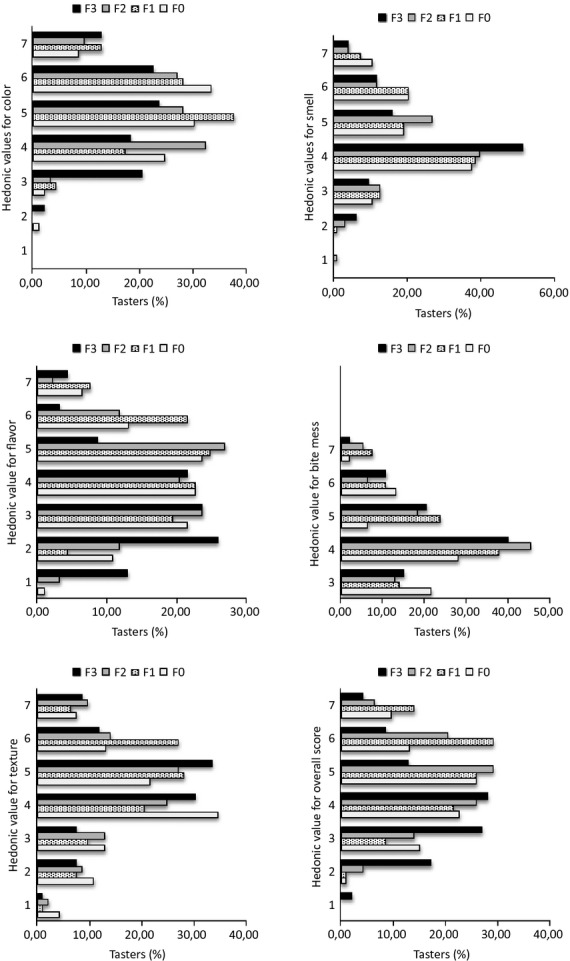
Distribution of providers by the evaluated characteristics: color, smell, flavor, bitterness, texture, and overall score, using hedonic scale.

In Table 1, the average of global impression of elaborated cassava flour for the following sensorial characteristics: color, smell, flavor, bitterness, texture, and overall score are presented. These average rates were assigned to the hedonic scale of 7 points. So in Figure 1, the distribution of testers according to the analyzed characteristics of elaborated cassava flour is represented. F_0_ (0% CPML), F_1_ (2.5% CPML), F_2_ (5% CPML), and F_3_ (10% CPML).

**Table 1:** Average grades of testers for sensorial characteristics of elaborated cassava flour

Characteristics	F_0_ (0% CPML)	F_1_ (2.5% CPML*)*	F_2_ (5% CPML)	F_3_ (10% CPML)
Color	5.18^ab^	5.28^a^	5.08^ab^	4.83^b^
Smell	4.80^a^	4.68^a^	4.95^a^	4.30^a^
Flavor	4.25^ab^	4.62^a^	4.00^b^	3.14^c^
Bitterness	4.10^a^	4.41^a^	4.08^a^	3.02^b^
Texture	4.57^ab^	4.73^a^	4.47^b^	4.41^ab^
Overall score	4.89^a^	5.15^a^	4.67^a^	3.75^b^

The same letters in the same line mean that there was not a significant difference (*P* > 0.05) Turkey test; 7, liked very much; 6, liked a lot; 5, liked; 4, indifferent; 3, did not like; 2, did not like a lot; 1, did not like very much.

The evaluations of color ranged from liked to liked a lot for flour F0, F1, and F2, but for the flour F3 they varied from indifferent and liked. It was noticed that there was no difference in the smell of the flour, the data of tasters varied from indifferent and liked. The flavor was pleasant for flour with 2.5% CPML, followed by flour with 0% CPML, 5% CPML, and 10% CPML. While regarding texture, this characteristic represented the impressions varying between indifferent and liked.

### Quantitative descriptive analysis

#### Descriptive terminology

Nine verbal terms were developed by the team of tasters to describe the similarities and differences among evaluated cassava flour samples. Each one of descriptors used was defined by the team of tasters and qualitative references were associated to each term (Table 2).

**Table 2:** List of descriptive terms, definitions, and references for each characteristic

Descriptive term	Definition	Reference
Color		
Whitish color	Characteristic color of raw manioc flour	Strong – wheat flour Weak – toasted manioc flour
Greenish color	Characteristic color of CPML	Strong – lemon gelatin powder No one – wheat flour
Flavor		
Manioc flavor	Characteristic manioc flavor	Strong – cooked manioc No one – distillated water
Bitter flavor	Characteristic bitter flavor presented in the caffeine solution	Strong – 0.06% caffeine solution in water No one – distillated water
Salty flavor	Flavor of saline solution	Strong – cookie cream cracker No one – distillated water
Characteristic flavor	Characteristic flavor of manioc flour	Strong – manioc flavor commercial No one – distillated water
Texture		
Lumpiness	Thick granules were noticed during the act of swallowing	Weak – wheat flour Strong – corn flour
Raw	Characteristic texture of wheat flour	Strong – row wheat flour Weak – toasted manioc flour
Smell		
Leave	Characteristic smell of fresh leave	Strong – 1 cut leave Weak – distillated water
Manioc	Characteristic smell of cooked manioc	Strong – cooked manioc Weak – distillated water

#### Sensorial profile of samples

**Figure 2:**
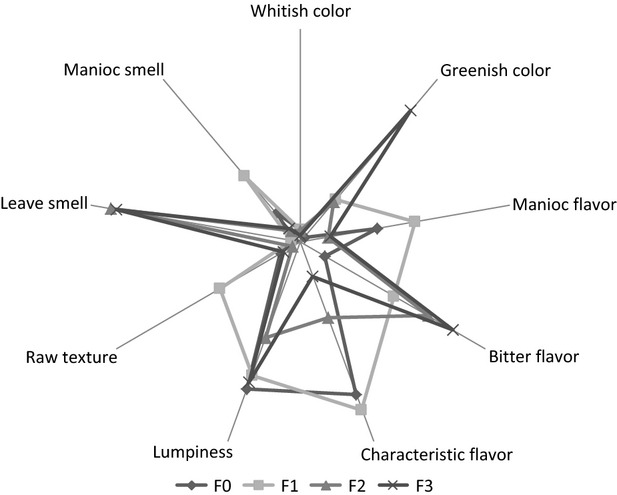
Configuration of quantitative descriptive analysis (QDA) for the characteristics of color, flavor, texture, and scent of elaborated cassava flour. F_0_ (0% CPML), F_1_ (2.5% CPML), F_2_ (5% CPML), and F_3_ (10% CPML).

The results show that the most whitish cassava flour was F_0_ (without addition of CPML), while the most greenish cassava flour was F_3_ with 10% CPML. Related to flavor, it was noticed that the less intensive flavor was in flour F_2_ and F_3_. On the other hand, the bitter flavor was more intensive in the samples with higher concentrations of CPML (F_3_, F_2_, F_1_, and F_0_). The characteristic flavor of cassava flour was more intensive in flour F_1_, F_0_, F_2_, and F_3_, respectively. For the characteristic of texture, lumpiness was more intensive in the sample F_0_, while the texture was less intensive in flour F_2_. The smell of the leaves was stronger in samples with higher concentrations of CPML. It was more noticeable in flour containing 5% CPML, while the smell of cassava was noticed better in samples F_1_, F_0_, F_3_, and F_2_, respectively (Fig. 2).

The results show that the most whitish cassava flour was F_0_ (without addition of CPML), while the most greenish cassava flour was F_3_ with 10% CPML. Related to flavor, it was noticed that the less intense flavor was in flours F_2_ and F_3_. On the other hand, the bitter flavor was stronger in the samples with higher concentrations of CPML (F_3_, F_2_, F_1_, and F_0_). The characteristic flavor of cassava flour was more intense in flour F_1_, F_0_, F_2_, and F_3_, respectively. For the characteristic of texture, there was more lumpiness in the sample F0, while the row texture was less in flour F_2_. The scent of a leave was stronger in samples with higher concentrations of CPML. It was detected better in flour with 5% CPML, while the scent of cassava was noticeably better in samples F_1_, F_0_, F_3_, and F_2_, respectively (Table 3).

**Table 3:** Average levels of sensorial descriptors of flour obtained by descriptive analysis

Characteristics	F_0_ (0% CPML)	F_1_ (2.5% CPML*)*	F_2_ (5% CPML)	F_3_ (10% CPML)
Whitish color	0.58^a^	0.55^a^	0.51^a^	0.34^a^
Greenish color	0.30^c^	2.59^b^	2.46^b^	8.06^a^
Manioc flavor	3.66^a^	5.49^b^	1.31^c^	1.43^c^
Bitter flavor	1.31^b^	5.09^a^	6.93^c^	8.28^c^
Characteristic flavor	7.64^c^	8.43^b^	3.79^ab^	1.70^a^
Lumpiness	7.34^a^	6.66^a^	4.81^b^	7.00^c^
Raw texture	1.09^a^	4.35^a^	0.44^b^	0.91^a^
Leaf scent	0.33^b^	0.44^a^	9.03^b^	8.78^b^
Manioc scent	1.76^b^	4.05^b^	0.70^a^	0.85^a^

The same letters in the same line mean that there was not a significant difference (*P* > 0.05) Turkey test.

The acceptance test has demonstrated that the cassava flour with 2.5% CPML was more appreciated by tasters without significant difference for flour with 0% and 5% CPML. This result is similar to data obtained by QDA. When more CPML is added to cassava flour, its characteristics become more distinct from that of the control flour, thus less preference by tasters.

## Conclusion

An addition of 2.5% CPML was preferred by tasters. However, there was no significant difference between control flour (without addition of CPML) and flour with 2.5% CPML and 5% CPML.

QDA has shown that higher concentrations of CPML in cassava flour negatively affected the greenish color, scent of the leaves, cassava flavor, and characteristics of cassava flour. However, the characteristics of flour with 2.5% and 5% CPML were close those of the control flour (without CPML addition), which means it is possible that flour prepared with CPML had better sensorial characteristics according to sensorial terms.
